# Alterations of protein expression of phospholamban, ZASP and plakoglobin in human atria in subgroups of seniors

**DOI:** 10.1038/s41598-019-42141-w

**Published:** 2019-04-04

**Authors:** Ulrich Gergs, Winnie Mangold, Frank Langguth, Mechthild Hatzfeld, Steffen Hauptmann, Hasan Bushnaq, Andreas Simm, Rolf-Edgar Silber, Joachim Neumann

**Affiliations:** 1 Institut für Pharmakologie und Toxikologie, Medizinische Fakultät, Martin-Luther-Universität Halle-Wittenberg, 06097 Halle/Saale, Germany; 2 Institut für Pathophysiologie, Medizinische Fakultät, Martin-Luther-Universität Halle-Wittenberg, 06097 Halle/Saale, Germany; 3 Institut für Pathologie, Medizinische Fakultät, Martin-Luther-Universität Halle-Wittenberg, 06097 Halle/Saale, Germany; 4 Klinik für Herz- und Thoraxchirurgie, Medizinische Fakultät, Martin-Luther-Universität Halle-Wittenberg, 06097 Halle/Saale, Germany

## Abstract

The mature mammalian myocardium contains composite junctions (*areae compositae*) that comprise proteins of adherens junctions as well as desmosomes. Mutations or deficiency of many of these proteins are linked to heart failure and/or arrhythmogenic cardiomyopathy in patients. We firstly wanted to address the question whether the expression of these proteins shows an age-dependent alteration in the atrium of the human heart. Right atrial biopsies, obtained from patients undergoing routine bypass surgery for coronary heart disease were subjected to immunohistology and/or western blotting for the plaque proteins plakoglobin (γ-catenin) and plakophilin 2. Moreover, the Z-band protein cypher 1 (Cypher/ZASP) and calcium handling proteins of the sarcoplasmic reticulum (SR) like phospholamban, SERCA and calsequestrin were analyzed. We noted expression of plakoglobin, plakophilin 2 and Cypher/ZASP in these atrial preparations on western blotting and/or immunohistochemistry. There was an increase of Cypher/ZASP expression with age. The present data extend our knowledge on the expression of anchoring proteins and SR regulatory proteins in the atrium of the human heart and indicate an age-dependent variation in protein expression. It is tempting to speculate that increased expression of Cypher/ZASP may contribute to mechanical changes in the aging human myocardium.

## Introduction

Aging represents an important risk factor for the development of cardiovascular diseases like heart failure. As the proportion of elderly people in the population increases, it is of growing importance to understand the aging process leading to changes in cardiac function and structure on molecular level. As numerous cellular pathways and proteins underlie changes during aging, we are focusing here on age dependent expression changes of a selection of cardiac proteins, responsible for Ca^2+^ regulation and cytoskeletal structural integrity.

For the structural integrity of the heart, junctional proteins are highly relevant^1^. Their mutations or altered expression levels can lead to cardiac malformations, development of heart failure and cardiac arrhythmias. In this work we focused on, firstly, plakophilin 2, secondly, plakoglobin which both are important linker molecules that connect members of the cadherin family of transmembrane proteins with the cytoskeleton and, thirdly, a structural protein of high cardiac relevance located to the Z discs called ZASP.

In the heart, solely the plakophilin isoform 2 is expressed^2^. Alterations in the levels of plakophilin 2 but also its mutation either directly lead to cardiac diseases or at least are correlated with cardiac diseases (reviewed in^3^). For example, mutations of plakophilin 2 are often found in patients with arrhythmogenic right ventricular dysplasia/cardiomyopathy (ARVD/C), which eventually can result in heart failure^4^. Moreover, genetic ablation of plakophilin 2 in mice is lethal and impairs the formation of appropriate cardiac ventricles^5^. Another junctional protein of interest in this context is plakoglobin. As plakophilin, plakoglobin is a linker that connects cadherins with the cytoskeleton^6,7^. It is of importance for proper cardiac function because, for instance, in homozygous knockout mice a rupture of cardiac ventricles was noted that was embryonically lethal^8^. In addition, mutations can lead to complex alterations in several organs including skin and heart, termed according to the location of high incidence as Naxos disease and various forms of ARVD/C^4,9,10^. The third structural protein we examined is called ZASP. It is the human homologue of Cypher. It interacts with alpha actinin at Z discs of striated muscle (heart and skeletal muscle)^11^. Mutations of Cypher/ZASP were noted in patients suffering from dilated cardiomyopathies (DCM)^12^. In mice, Cypher knockout leads to severe myopathies^13,14^. The cardiac-specific knockout induced a severe form of DCM with disrupted cardiomyocyte ultrastructure and decreased cardiac function, which led to premature death.

Other proteins whose expression is correlated with cardiac dysfunction are located to the sarcoplasmic reticulum (SR) and they are highly important for maintaining Ca^2+^ homeostasis. In the myocardium, Ca^2+^ induced Ca^2+^-release from the sarcoplasmic reticulum via activation of ryanodine receptors (RyRs) is the main mechanism of cardiac excitation-contraction coupling^15,16^. The ensuing increase in intracellular Ca^2+^ concentration is responsible for muscle contraction^15^. For relaxation, Ca^2+^ is mainly removed from the cytosol by the action of SR Ca^2+^-ATPase (SERCA) into the SR. The affinity of SERCA for Ca^2+^ is regulated by phospholamban (PLB) located in the SR. The main cardiac Ca^2+^ storage protein of the SR is calsequestrin 2 (CSQ) that is located in the lumen of the SR. RyR and CSQ are situated in the junctional SR whereas PLB and SERCA are seen in the free SR. The action of β-adrenoceptor agonists is mediated, at least in part, by PLB as knockout or mutation of PLB attenuate the cardiac effects of β-adrenergic catecholamines and can lead to cardiac morphological changes and dysfunction^17^. Upon aging, the function of the SR, as judged from animal studies, is impaired. For instance, on aging not only the expression of β-adrenoceptors is decreased, but also the effectiveness to release Ca^2+^ from the SR is attenuated^18^. This correlates with and is explained by, at least in part, a reduced expression of SERCA (rat^19^, for review see^20^). A progress report of this work has appeared in abstract form^21^.

## Results

**Figure 1:**
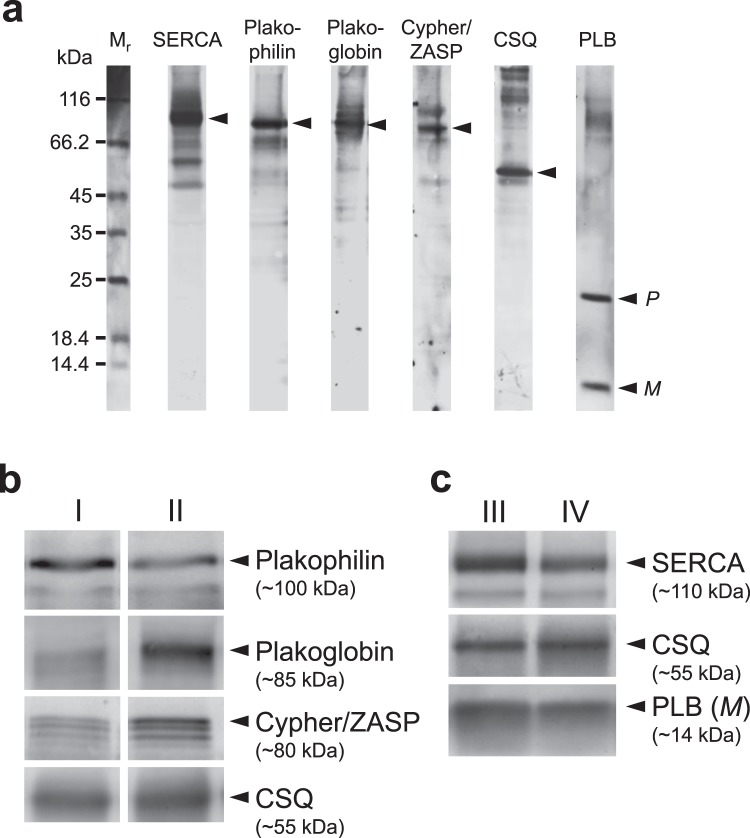
Western blotting. Homogenates from human cardiac atria were subjected to gel electrophoresis and Western blotting as described in the Methods section. (**a**) Detection of whole lanes is given for one exemplary patient to demonstrate quality of antibodies used in this study. A typical marker lane (Mr) is shown on the left side. *P* and *M* are the pentameric and monomeric mobility forms of phospholamban. (**b)** Representative Western blots of plakophilin, plakoglobin and Cypher/ZASP. Calsequestrin (CSQ) was studied as cardiomyocyte-specific loading control. (**c)** Representative Western blots of three SR proteins (CSQ as cardiomyocyte-specific loading control; sarcoplasmic reticulum Ca^2+^ ATPase, SERCA; phospholamban, PLB). Apparent molecular weights are added in kDa (kilo Dalton). (**b**,**c**) Left hand lanes (I, III) are from a younger patient, right hand lanes (II, IV) are from an older patient.

**Figure 2:**
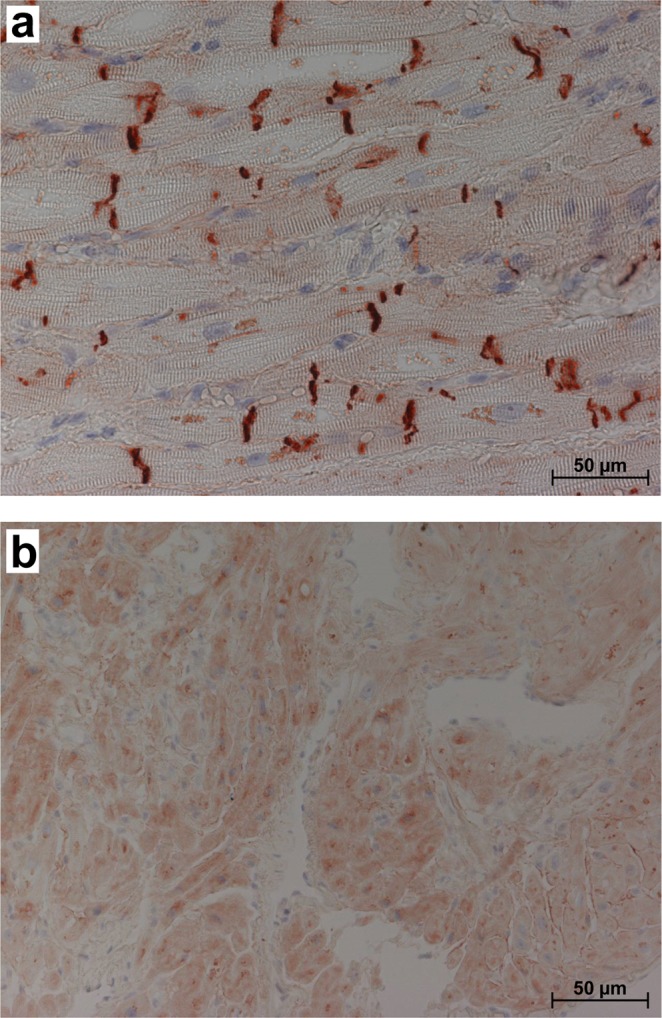
Immunohistology. Samples obtained from human right atrium were fixed and stained for (**a**) plakoglobin or (**b**) Cypher/ZASP. The primary antibodies were directed against the antigen and a secondary antibody gave rise to a color reaction as described in the Methods section.

**Figure 3:**
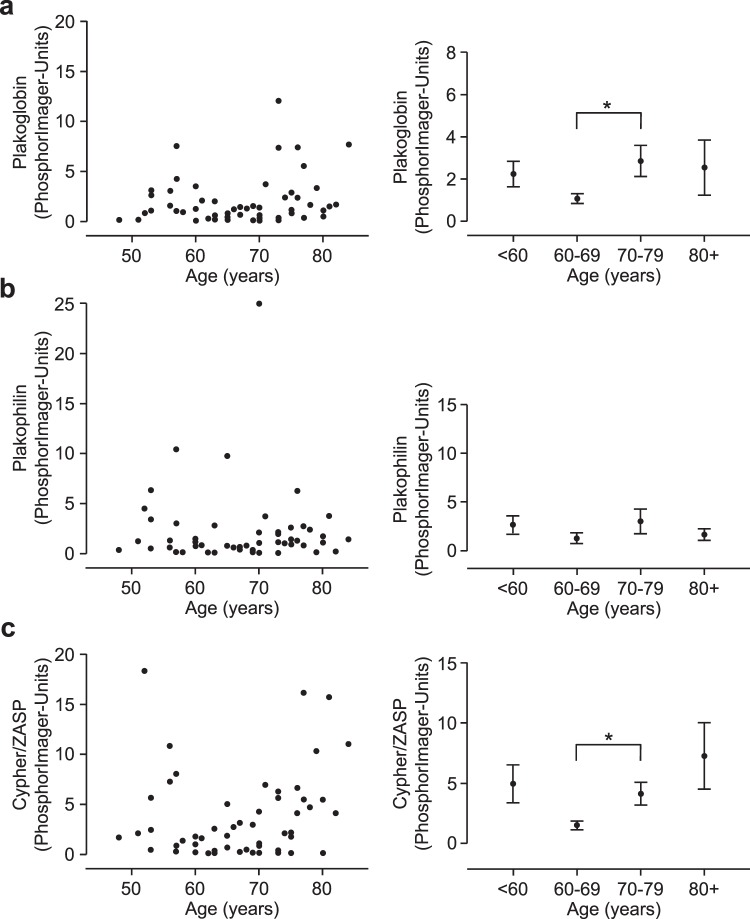
Correlation of (**a**) plakoglobin, (**b**) plakophilin, and (**c**) Cypher/ZASP expression in human right atrium (left). Ordinates are protein expression (normalized to CSQ, in arbitrary phosphorImager units) versus age (abscissae). Data on the right hand side give means of ten year intervals ± SEM. *Indicates a significant difference between age groups 70–79 vs. 60–69. N(total) = 53, N(<60) = 12, N(60–69) = 17, N(70–79) = 19, N(80+) = 5.

**Figure 4:**
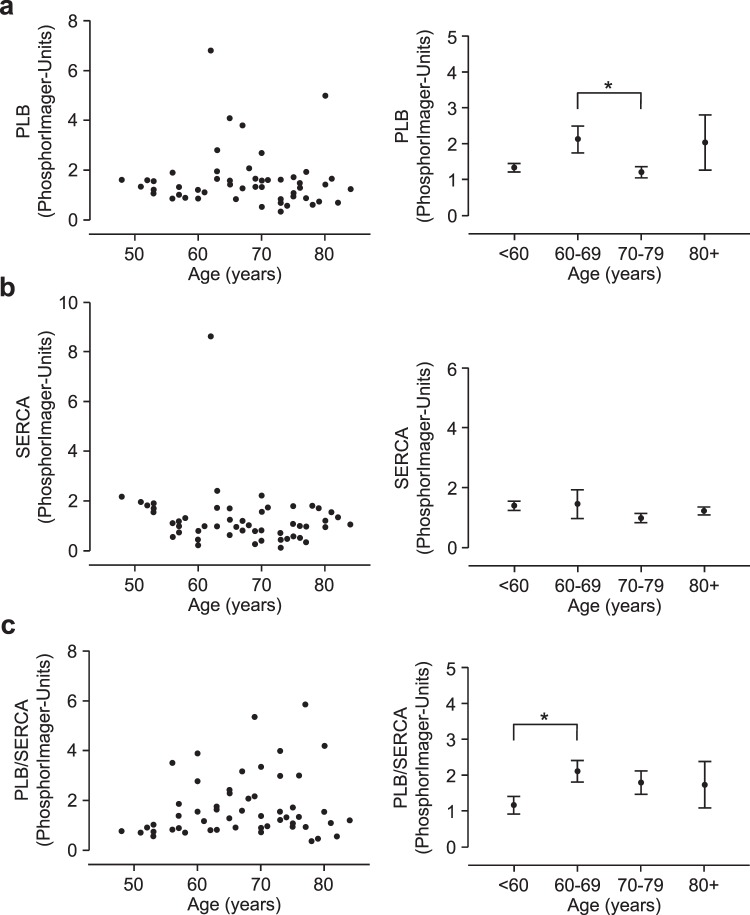
Correlation of (**a**) PLB, (**b**) SERCA, and (**c**) the ratio of PLB and SERCA (PLB/SERCA) expression in human right atrium (left). Ordinates are protein expression (normalized to CSQ in arbitrary phosphorImager units) versus age (abscissae). Data on the right hand side give means of ten year intervals ± SEM. *Indicates a significant difference between age groups 70–79 vs. 60–69 (**a**) or below 60 vs. 60–69 (**c**). N(total) = 53, N(<60) = 12, N(60–69) = 17, N(70–79) = 19, N(80+) = 5.

Antibody specificity was first validated using Western blotting (Fig. 1a) and immunohistochemistry. Homogenates were prepared from frozen human atrial samples and subjected to Western blotting. Representative Western blots for plakoglobin, plakophilin and Cypher/ZASP are presented in Fig. 1b. Similar experiments were done with PLB and SERCA and are depicted in Fig. 1c. In Supplementary Fig. S1, examples of typical original Western blots are presented. These examples visualize one problem of the study, a high scattering of the Western blot experiments. This point will be addressed in the discussion. Cardiac CSQ was used as a cardiomyocyte-specific marker to normalize the protein loading to the cardiomyocyte content. The suitability of CSQ as housekeeping gene was tested: there was no age dependent expression of CSQ and CSQ expression correlated with Ponceau S protein staining of the blotting membranes (Supplementary Fig. S2). All antibodies showed specific labeling of proteins with the expected molecular weights (about 85 kDa for plakoglobin, 100 kDa for plakophilin 2 and 80 kDa for Cypher/ZASP). Cypher/ZASP revealed several bands below 80 kDa as expected since three long splice variants (ZASP-1, -2 and, -5) have been described (Fig. 1b)^12^. We also tested the antibodies against the junctional proteins in immunohistochemistry. Figure 2a shows the localization of plakoglobin, which is readily apparent at the disci intercalares, as expected for this junctional protein. In contrast, Cypher/ZASP, which is expected to localize near Z-bands, is not concentrated at the longitudinal border of each cell but within each cell, consistent with the published literature (Fig. 2b). Since these preliminary tests revealed the specificity of the antibodies, additional samples were subjected to Western blotting for a quantitative evaluation of expression patterns. Again, CSQ was used as an internal cardiomyocyte-specific loading control. Using these precautions we plotted age of patient versus protein expression (Fig. 3 left hand side). There was much fluctuation in the data. As a next step we clustered the data into ten year intervals. The results are shown on the right hand side of Fig. 3. There were no significant differences between the groups with respect to plakophilin. Plakoglobin 2 levels were significantly lower in the age group between 60 and 69 compared to those between 70 and 79. Likewise Cypher/ZASP values were significantly lower in the age groups of patients between 60 and 69 compared to those between 70 and 79. The expression of PLB and SERCA related to CSQ are depicted in Fig. 4 in a similar way. Of note, PLB expression was lower in an older group (70–79), than in a younger group (60–69). Furthermore, it is reasonable to assume that PLB and SERCA are functionally antagonistic in regard to Ca^2+^ pumping into the SR. Hence, frequently the quotient of these proteins is presented. This was done in Fig. 4 (bottom). It is noteworthy that here the group below 60 showed less expression than that at 60–69. As clinically data are often presented in the age categories <65, 65–74, and >74 we, therefore, rearranged the clustered data into these three intervals (Supplementary Fig. S3). The overall results were comparable: the PLB/SERCA ratio was higher in the 65–74-year group compared to younger patients (<65) and plakoglobin as well as Cypher/ZASP expression was increased in older patients (>74) (Fig. S3).

In Table 1, correlation for parameters, which we regarded as relevant, are shown. Notably, there was only a trend (p < 0.34) towards a negative correlation between age and SERCA expression. No correlation with age gained significance. Between SR proteins, there was a strong positive correlation between PLB and SERCA expression. Between junctional proteins a high positive correlation between Cypher/ZASP and plakoglobin expression was noteworthy. As concerns drug treatment, we noted negative correlations between ASS (acetylsalicylic acid) administration and phospholamban and plakoglobin expression. Finally, there was a positive correlation between ACE inhibitor/AT1 antagonist and plakoglobin expression.

**Table 1:** Linear regression model.

	PLB		SERCA		PLB/SERCA		PKP		Plako		ZASP	
	β	p	β	p	β	p	β	p	β	p	β	P
Age	0.07	0.36	−0.1	0.34	0.04	0.66	−0	0.97	0.1	0.41	0.1	0.48
EF	0.05	0.52	−0.1	0.12	−0	0.87	−0	0.93	−0.1	0.39	−0	0.75
Statins	−0	0.8	0.05	0.52	0.11	0.28	0	1	0.08	0.541	−0.2	0.18
CCS	−0.1	0.46	0.02	0.84	−0.1	0.21	−0.2	0.2	0.05	0.705	0.23	0.12
ASS	**−0**.**2**	**0**.**04**	0.03	0.74	0.11	0.34	0.05	0.81	**−0**.**4**	**0**.**002**	0.01	0.95
ACE or AT1	0.1	0.25	−0	0.9	−0	0.72	0.04	0.83	**0**.**3**	**0**.**017**	−0.1	0.4
PLB			**0**.**78**	**0**	**0**.**79**	**0**	0.04	0.91	−0.3	0.209	−0.2	0.54
SERCA	**0**.**81**	**0**			**−0**.**9**	**0**	−0.1	0.7	−0.1	0.728	0.17	0.52
PLB/SERCA	**0**.**51**	**0**	**−0**.**6**	**0**			−0.3	0.29	−0	0.954	0.04	0.84
PKP	0.01	0.91	−0	0.7	−0.1	0.29			0.01	0.962	0.14	0.29
Plako	−0.1	0.21	−0	0.73	−0	0.95	0.01	0.96			**0**.**34**	**0**.**04**
ZASP	−0.1	0.54	0.06	0.52	0.02	0.84	0.2	0.29	**0**.**28**	**0**.**043**		

Protein expression data were normalized to CSQ expression.

β, standardized regression coefficient; ACE, Angiotensin-converting enzyme antagonist; ASS, acetylsalicylic acid; AT1, angiotensin receptor type 1 antagonist; CCS, Canadian Cardiovascular Society angina class; EF, ejection fraction; PKP, plakophilin; Plako, plakoglobin; PLB, phospholamban; SERCA, sarcoplasmic reticulum Ca^2+^-ATPase; ZASP, Cypher/ZASP.

## Discussion

Atrial tissue from patients undergoing bypass surgery due to coronary heart disease was studied in the present work. All patients included in this study were on β-adrenoceptor blocker therapy. β-adrenoceptor-blockers can alter many of the biochemical parameters studied here. For instance, the expression of plakoglobin is altered, in animal studies, following β-adrenoceptor-blocker therapy^22^. Several studies on gene expression during aging in animal models (for review see e.g.^23^) and in human tissue also using gene expression arrays (for review^24^) have been published. In humans, 162 candidate gene products correlating with heart failure were identified. However, only mRNA for methionine tRNA synthase correlated with age^24^. In non-failing human hearts, only two transcripts correlated with age, for instance the abundance of metallothionein 1 L increased with age^24^. These data were obtained in ventricular tissue and on mRNA levels, whereas we studied atrial tissue and protein expression.

### Cytoskeletal proteins

According to the published literature, plakophilin is present solely in disci intercalares of cardiomyocytes (bovine heart^2^). Lack of plakophilin 2 led to reduced expression of important calcium regulatory proteins and to disruption of intracellular calcium homeostasis^25^. From these data it was suggested that plakophilin 2 mutations in humans might cause arrhythmias even in the absence of structural abnormalities^25^. In this regard, some forms of ARVD/C are accompanied by mutations of plakophilin 2^26^. But no age dependent morphological changes of ARVD/C were noted^27^. Also here, plakophilin expression was independent from age or drug application (limited to the drugs listed in Table 1) of the patients studied.

With respect to age, a similar situation was found for plakoglobin also known as γ-catenin. The immunohistological localization of plakoglobin is in line with previous work. It was seen solely in the disci intercalares but not in the cytosol or the cell nucleus^28^. As concerns the function of plakoglobin, mutations of plakoglobin are associated with cardiac diseases such as ARVD/C^26,29^. Moreover, a reduced expression of plakoglobin has been reported in the myocardium of patients with ARVD/C but a causal link between plakoglobin expression and ARVD/C remained unknown^30^. To our knowledge, age-dependent changes in cardiac plakoglobin have not yet been investigated. In the patient collective we studied, plakoglobin expression seems to increase with age at least when looking to distinct age groups. Nevertheless, no correlation with age was found when comparing all patients. Therefore, the results have to be interpreted carefully, as the number of old patients is small and especially in this group of patients, the scattering of protein expression is high. Interestingly, expression of plakoglobin seems to be influenced by several drugs. A negative correlation was found for ASS that is known to change expression of pro- and anti-inflammatory genes^31,32^. In contrast, a positive correlation was found for ACE or AT1 antagonists. Maybe this observation reflects the inhibition of fibroblast proliferation by these drugs as demonstrated for example by Yu *et al*.^33^.

A similar age related expression pattern as noted for plakoglobin was found for Cypher/ZASP. The immunohistological localization of Cypher/ZASP was in line with previous work, but in contrast to earlier work our data were obtained in atrium^34^. The protein is not (using our detection methods) present in the nucleus of cell or the disci intercalares but mainly and diffusely in the cytosol. The cardiac specific isoforms of Cypher/ZASP (also called Oracle) are 1c, 2c, and 3c^35^. In some of our Western blots, the resolution was high enough to separate distinct bands possibly representing splice variants (Supplementary Fig. S1). In the case of multiple bands, we used the sum of signals for quantification. Cypher/ZASP is located in the Z-disc of cardiac muscle and physically interacts with proteins of diverse functions, like regulation of glucose metabolism^34^. A pivotal role of Cypher/ZASP for maintaining adult cardiac structure and cardiac function was demonstrated in mice and Cypher/ZASP mutations in patients with DCM underline the importance of this protein^14^. Moreover, Cypher/ZASP specifically interacted with the regulatory subunit II of protein kinase A and calcineurin. Therefore, Cypher/ZASP was suggested to act as A-kinase anchoring protein expanding its role to the regulation of the phosphorylation and thus function of sarcomeric proteins like channels and myofilament proteins^36^. In this context, any evidence for an age-related expression would be of interest. Here, it seems that Cypher/ZASP expression increases with age, but only when comparing distinct age groups. Accordingly, Cypher/ZASP expression correlated positively with plakoglobin expression.

Therefore, we speculate cautiously that increased expression of cytoskeletal proteins may reflect an adaptation of cardiac myocytes to maintain function with increasing age. Fittingly, when decreased expression or function (e.g. mutated proteins) of plakoglobin or Cypher/ZASP was reported, it was associated with cardiac diseases.

### Calcium regulatory proteins

As demonstrated for cytoskeletal proteins neither for PLB and SERCA nor for the PLB/SERCA quotient a correlation with age was found in the collective of patients studied. In heart failure, there is scant evidence for PLB downregulation. While the mRNA is usually reported to be reduced, most studies have shown that PLB protein is not altered. Cain *et al*. noted a decrease of pentameric PLB but not monomeric PLB^37^. However, we disagree with their argument for doing these experiments^37^. They argued that the monomeric form of PLB is a better predictor of PLB induced inhibition of SERCA. They froze their samples at −70 °C^37^. Under these conditions it is well known that PLB will time dependently aggregate again to its pentameric form^38^. Hence, this might lead to artifacts. Therefore, we boiled our samples on purpose to convert all PLB to the monomeric form, in order to rule out this possible bias. Moreover, we think that our patient population is better defined and larger (see next paragraph).

SERCA mRNA is down regulated in heart failure. There is a controversy whether or not SERCA protein levels are decreased^39^ or not^40^. However, the function of SERCA is consistently reduced, possibly due to an altered ratio of PLB to SERCA. For mRNA expression levels, studies from aging rat hearts or human hearts reported a down regulation of SERCA. Others noted a down regulation of SERCA on protein level upon aging in human atrial preparations^37^. Looking closely at the clinical characteristics of those patients several differences between the patients in^36^ and our study are apparent, which may alone or together explain our divergent results: firstly, we used much higher numbers of patients, they used 12 patients^37^, and we studied 60 patients. They used male and female patients; we only used male patients. In their study some patients were hypothyroid and some were not treated with β-adrenoceptor blockers^37^. Any of these differences alone might explain theoretically the discrepancies. More specifically, we have reported on a tendency of an increase in SERCA in right atrium of patients with latent hypothyroidism^41^.

Whilst expression and/or function of SERCA and PLB are commonly decreased in the course of cardiac diseases, any changes during aging are still part of a controversial discussion. Here, we found no convincing evidence for age-dependent regulation of SERCA or PLB expression in human right atrium.

### Study limitations

One drawback of the present study can be seen in the fact that we have only studied diseased myocardium. However, non-failing myocardium is not available in our institution and such data, perhaps obtained via non-invasive methods, are awaited with interest. Moreover, due to lack of tissue, we have not been able to study ventricular tissue. Furthermore, one can ask how expression levels are in childhood or young adults. The latter studies over the full age range of humans might reveal changes occurring of many decades and might be helpful to discover much greater changes in protein expression. Another serious limitation of the present study was that all patients obtained several drugs and we cannot exclude that drug effects obscured some of the changes that are present in the aging myocardium. Indeed, in multiple correlations drug effects (e.g. ASS) were visible in the samples studied here. It was clinically required to treat patients with drugs, following current guidelines. At least, we included only patients on β-adrenoceptor-blocker therapy to exclude this as a confounding variable. In addition, we excluded severely ill patients (NYHA IV) because that was expected to bias our result, as many gene alterations are known in end stage heart failure. Indeed, ejection fractions were in the normal range arguing for the absence of (systolic) heart failure in the study patients.

Regrettably, we cannot provide data on cardiac ageing in non-diseased myocardium as it is practically and ethically not feasible to get atrial samples of non-diseased yet. We have included patients who suffer from coronary heart disease but have no severe clinical features of heart failure: patients are in NYHA I or II.

It can be asked why we used calsequestrin for normalization of protein expression. Calsequestrin has the advantage of being a protein exclusively expressed in cardiomyocytes (as far as we know the first report in the heart^42^). We have generated CSQ2 KO (knock-out) mice^43^. Indeed, in these mice we detected no signals in atrium of CSQ2 KO mice^43^. In that paper, we ran into the problem how to make sure that we had equal loading of the lanes and therefore normalized to glycerinaldehyd-3-phosphate-dehydrogenase (GAPDH)^43^ hence, we know this problem and have used GAPDH as loading control, but only when calsequestrin was absent. A similar thinking is seen in the study of Herraiz-Martínez^44^. There, in Fig. 4 the authors cleverly reported the ratio of CSQ2/GAPDH. Indeed, GAPDH is a well-accepted housekeeping protein: it is present in cardiomyocytes but abundantly also in non-myocytes. Hence, in the course of ageing or the underlying coronary heart diseases, a proliferation of non-myocytes might have occurred (for instance, by a low hardly detectable level of increase in the number of fibrocytes), and then one may erroneously normalize one’s proteins of interest to the amount of a mixture of cardiomyocytes and non-cardiomyocytes. Hence, we state this point as a limitation of our study but think for the time being that to measure against CSQ2 is an alternative valid approach if one wants to refer to the protein expression with regard to cardiomyocytes like in the present study.

Finally, it can be asked why there was a lot of scatter in the protein expression data, much more than obtained with the same biochemical methods in aging animal hearts. From previous work in animal tissue: rat, guinea pig, dog, and mice heart, we never saw such a higher scatter of data. We took all the precautions and controls we could think of and were feasible in our lab to look for systematic mistakes on our part. We did several controls: for instance processing human and animal cardiac samples on the very same day with the same equipment, the same freshly made solutions and the very same batch of antibody. Our hunch is that is has something to do with the patients. Perhaps confounding cardiac diseases, perhaps not yet manifest maladies of patients, confounding drug treatment, patients taking recreational drugs unbeknownst to their attending physicians. Otherwise, the scatter may simply mean that either cardiac aging entails many, at present, hidden variables or expression of these proteins is generally subject to certain variability in human hearts.

## Conclusions

We tentatively conclude that junctional proteins may form a part of the aging process in the human heart.

## Methods

### Patients

From animal studies it is known that the expression of Ca^2+^ regulatory proteins is affected by high levels of β-adrenergic stimulation^45^. Others and we have shown that hypothyroidism as well as hyperthyroidism can affect the proteins of interest in our study (e.g.^41^): Moreover, gender affects expression of Ca^2+^ regulatory proteins (e.g. in humans^46^): Therefore, to minimize scatter in data, our inclusion criteria were male sex and treatment with β-adrenoceptor blockers metoprolol, bisoprolol or carvedilol. Exclusion criteria were treatment of any thyroid disorder, NYHA class > III, ejection fraction under 35% and treatment with more than 12 different drugs (which we noted in one patient undergoing surgery). Medications that were included in the correlations analysis were (in brackets the percentages of all patients are given): Statins (74%), ASS (38%), ACE-inhibitors (38%), AT1 receptor blockers (4%). For further analysis, we pooled patients on ACE-inhibitors and AT1 receptor blockers (41%). The means NYHA class was 2.00 ± 0.41, the mean left ventricular ejection fraction was 62 ± 11.3%, the mean CCS was 2.00 ± 0.58. The detailed patient characteristics are listed in the Supplementary Table S1. Patients underwent bypass surgery in our institution due to coronary heart disease and in the course of this intervention right atrial tissue was obtained and quickly frozen to the temperature of liquid nitrogen and kept in a −80 °C freezer until biochemical analysis. All patients were between 48 and 84 years old (median value 68, mean value 67). This study protocol conforms to the ethical guidelines of the 1975 Declaration of Helsinki and has been approved by the ethics committee of the medical faculty of the Martin Luther University Halle-Wittenberg (hm-bü 04.08.2005) and patients gave informed consent.

### Western blot analysis

For Western blot analysis, tissue lysates were prepared and 4x strength SDS sample buffer containing 250 mM Tris HCl (pH 6.8), 20% (wt/vol) SDS, 40% (vol/vol) glycerol, 1.2% (wt/vol) dl-dithiothreitol, and 0.004% (wt/vol) bromophenol blue was added^43^. The samples were solubilized for 10 min at 95 °C. Aliquots of 55 µg (resp.40 µg) protein were loaded per lane and gels were run using 10% polyacrylamide separating gels. Proteins were electrophoretically transferred to nitrocellulose membranes in 50 mM sodium phosphate buffer (pH 7.4) 180 min at 1.5 A at 4 °C. We evaluated the quality of the transfer by reversible Ponceau S staining of the membranes. Then, membranes were treated with TRIS-buffered saline containing 5.0% non-fat dry milk powder and 0.1% Tween 20 for 60 min at room temperature followed by incubation with primary antibodies overnight at 4 °C. Subsequently, alkaline phosphatase labeled secondary antibodies were used and bands were detected using enhanced chemofluorescence (ECF, GE Healthcare, Munich, Germany). Fluorescent bands were visualized in a STORM PhosphorImager and quantified using the ImageQuaNT software (GE Healthcare, Munich, Germany). Following primary antibodies were used: mouse monoclonal anti plakoglobin (dilution 1:1000; Sigma-Aldrich, Munich, Germany), mouse monoclonal anti plakophilin 2 multi-epitope cocktail (dilution 1:150; Progen Biotechnik, Heidelberg, Germany) and mouse monoclonal anti Cypher 1 (dilution 1:1000; BD Transduction Laboratories, Heidelberg, Germany), rabbit polyclonal anti calsequestrin (dilution 1:2500; Acris Antibodies, Hiddenhausen, Germany), mouse monoclonal anti SERCA (dilution 1:3000 or 1:10000; kindly provided by L.R. Jones, Indianapolis, IN, USA) and mouse monoclonal anti PLB (dilution 1:2000; A-1, Badrilla, Leeds, UK). Secondary antibodies (Sigma-Aldrich, Munich, Germany) were all diluted 1:1000 and incubated for 2 hours at room temperature. First, we studied linearity of loaded protein versus antibody detection in typical samples. We noted a linear range from 10 to 120 µg (data not shown) for all antibodies studied. In all subsequent experiments we used those amounts of antigen (that is human atrial homogenates) that were within these linear ranges. On each gel, a reference sample was run that was used to compare between gel runs^43^. The specificity of the antibodies was tested in numerous previous studies. These studies were done in isolated cardiomyocytes (mouse, guinea pig: e.g.^47^ or in multicellular preparations from atrial tissue (mouse^48^, rat^45^, human heart^40^). We have checked the specificity over the years in various ways, also be infecting cells with adenovirus coding for SERCA. As another control it is accepted that SERCA 2a is present in the heart, whereas SERCA 2b is present mainly in the smooth muscle cell of many human tissues. In the past, we had the opportunity to test the antibody in the human esophagus, which contains both SERCA 2a and 2b, which run on gels at different molecular weight. Doing this we would find in parallel gel runs in human heart samples one band and in human esophagus samples only one band. (Fig. 1 in^49^). Evidence for the specificity of our CSQ-2 antibody comes from another of our studies: in CSQ-2 KO mice, no signal was detected in the atrium with this CSQ antibody^43^.

### Immunohistochemistry

Standard immunohistochemistry was performed as follows: 4-µm paraffin-embedded tissue sections were prepared and for antigen retrieval, slides were boiled in 10 mM citrate buffer (pH 6.0) for 25 minutes in a preheated (95–100 °C) pressure cooker. After that, slides were placed at room temperature and allowed to cool down for 20 min. Sections were covered with primary antibodies to plakoglobin and Cypher (dilution 1:400) at 37 °C for 30 min. followed by rinsing in PBS (phosphate buffered saline). After 15 min of incubation with the biotinylated secondary antibody at room temperature (Zytochem Plus HRP Kit; Zytomed Systems GmbH, Berlin, Germany), the slices were rinsed in PBS, incubated 15 min with streptavidin-HRP-conjugate at room temperature and again rinsed in PBS. Subsequently, HRP was developed with Vector^®^ AEC chromogen substrate (Biozol, Eching, Germany) for 10 min. Finally, the slices were rinsed in distillated water and counterstained with hematoxylin. Negative controls were performed by omission of primary antibodies or substitution of primary antibody by rabbit IgGs at the same final concentration^43^. Control incubations resulted in a lack of immunostaining. Images shown are representative of at least three independent experiments from different patients which gave similar results.

### Statistics

Data are where indicated presented as means ± SEM. Comparisons were evaluated using one-way ANOVA followed by Bonferroni’s test for multiple-group comparisons using GraphPad Prism version 5.03 for Windows, GraphPad Software, San Diego California USA, www.graphpad.com. Gaussian distribution was checked by the implemented test (D’Agostino & Pearson) of Graphpad Prism. Moreover, regression analysis was performed for the following parameters using the SPSS 17 package: Medication (taking of statins, acetylsalicylic acid, ACE-inhibitors), PLB, SERCA, Cypher/ZASP, Canadian Cardiovascular Society (CCS) angina class, NYHA stage and age. A value of *p* < 0.05 was considered statistically significant.

## Supplementary information

Supplementary data
