# Supplementary material for: Alterations of protein expression of phospholamban, ZASP and plakoglobin in human atria in subgroups of seniors
Source: Sci Rep. 2019 Apr 4;9:5610. doi: 10.1038/s41598-019-42141-w (PMC6449388; doi:10.1038/s41598-019-42141-w)
Supplement: Supplementary file 1 — Supplementary data [file 41598_2019_42141_MOESM1_ESM.pdf]

Alterations of protein expression of phospholamban, ZASP and plakoglobin in human atria in subgroups of seniors.

Ulrich Gergs[a*], Winnie Mangold[a], Frank Langguth[a], Mechthild Hatzfeld[b], Steffen Hauptmann[c], Hasan Bushnaq[d], Andreas Simm[d], Rolf-Edgar Silber[d], Joachim Neumann[a]

[a]Institut für Pharmakologie und Toxikologie, Medizinische Fakultät, Martin-Luther-Universität Halle-Wittenberg, 06097 Halle (Saale), Germany

[b]Institut für Pathophysiology, Medizinische Fakultät, Martin-Luther-Universität Halle-Wittenberg, 06097 Halle (Saale), Germany

[c]Institut für Pathology, Medizinische Fakultät, Martin-Luther-Universität Halle-Wittenberg, 06097 Halle (Saale), Germany

[d]Klinik für Herz- und Thoraxchirurgie, Medizinische Fakultät, Martin-Luther-Universität Halle-Wittenberg, 06097 Halle, Germany

Short title: Protein expression in human atria

*Corresponding author: Ulrich Gergs, Institut für Pharmakologie und Toxikologie, Medizinische Fakultät, Martin-Luther-Universität Halle-Wittenberg, Magdeburger Str. 4, 06112 Halle (Saale), Germany

Tel.: +49-345-557 4093, Fax: +49-345-557 1835, E-mail: ulrich.gergs@medizin.uni-halle.de

Supplementary Figures

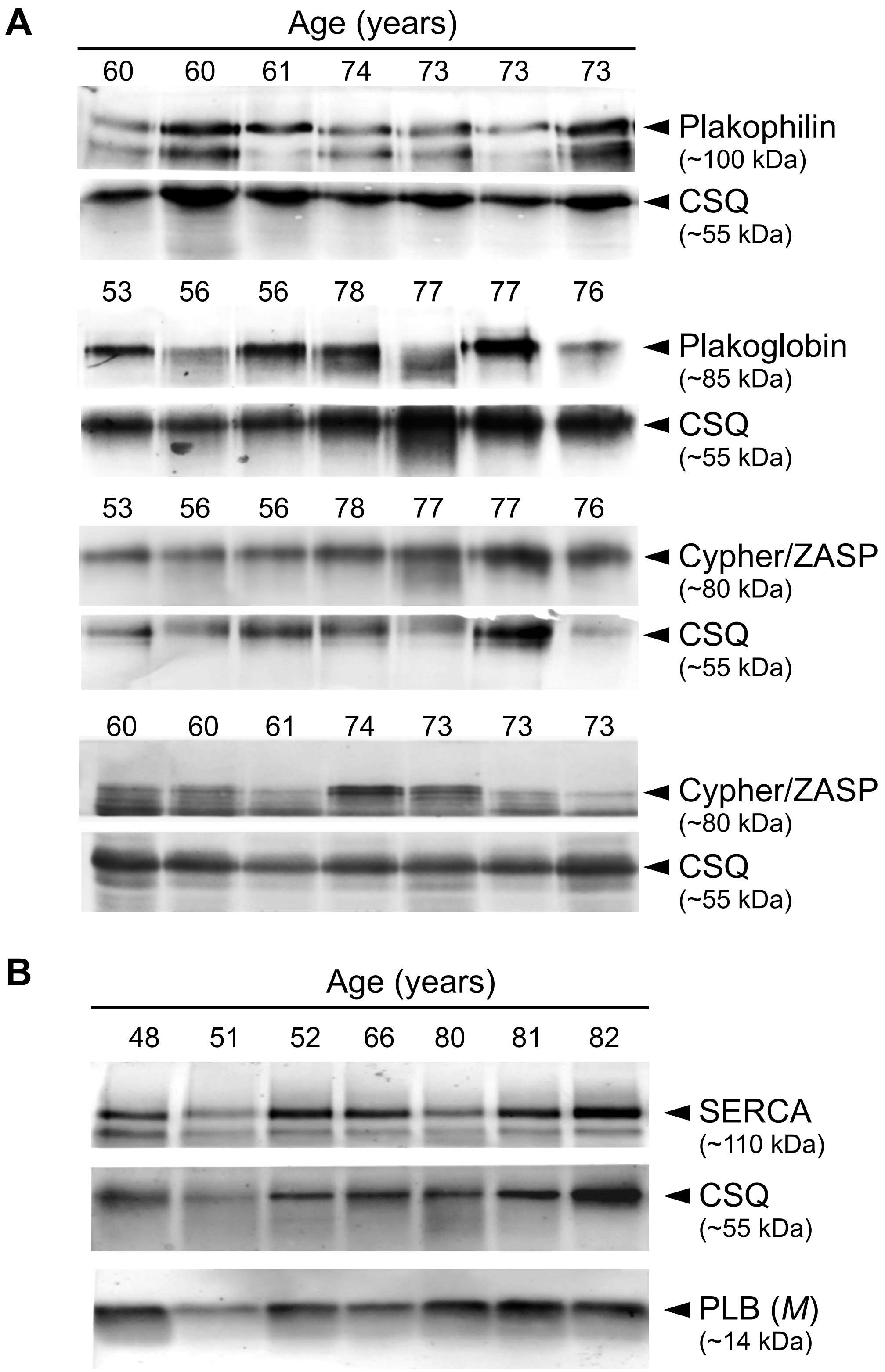

Figure S1. Examples of original Western blotting experiments. Expression of (**A**) plakophilin, plakoglobin, and the Z-band protein cypher 1 (Cypher/ZASP) and of (**B**) sarcoplasmic reticulum Ca^{2+}-ATPase (SERCA) and phospholamban (PLB) in human right atrium from patients of different age as indicated. Calsequestrin 2 (CSQ) expression was used as loading control. The corresponding CSQ blots are shown. These examples demonstrate the scattering of the Western blot experiments as discussed in the section “study limitations”.

Supplementary Figures

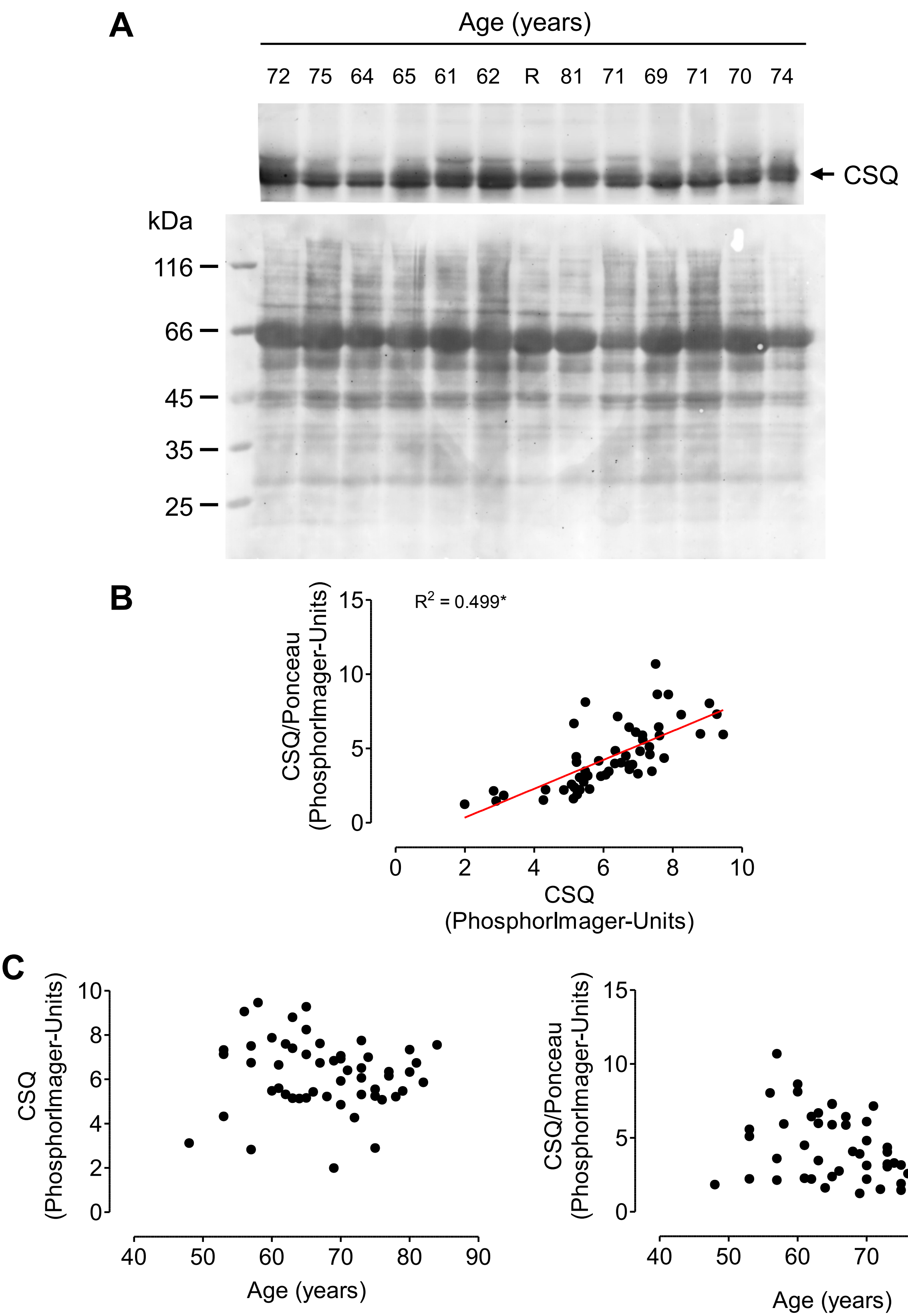

Figure S2. Expression of cardiac calsequestrin 2 (CSQ) in human right atrium. (**A**) One representative Western blot experiment for CSQ and the corresponding Ponceau S staining of the membrane. (**B**) Correlation of CSQ with CSQ normalized to Ponceau S staining. (**C**) Correlation of CSQ and normalized CSQ with age demonstrating no age-dependent CSQ expression in human right atrium. * indicates a significant correlation ($p<0.05$). N(total) = 53.

Supplementary Figures

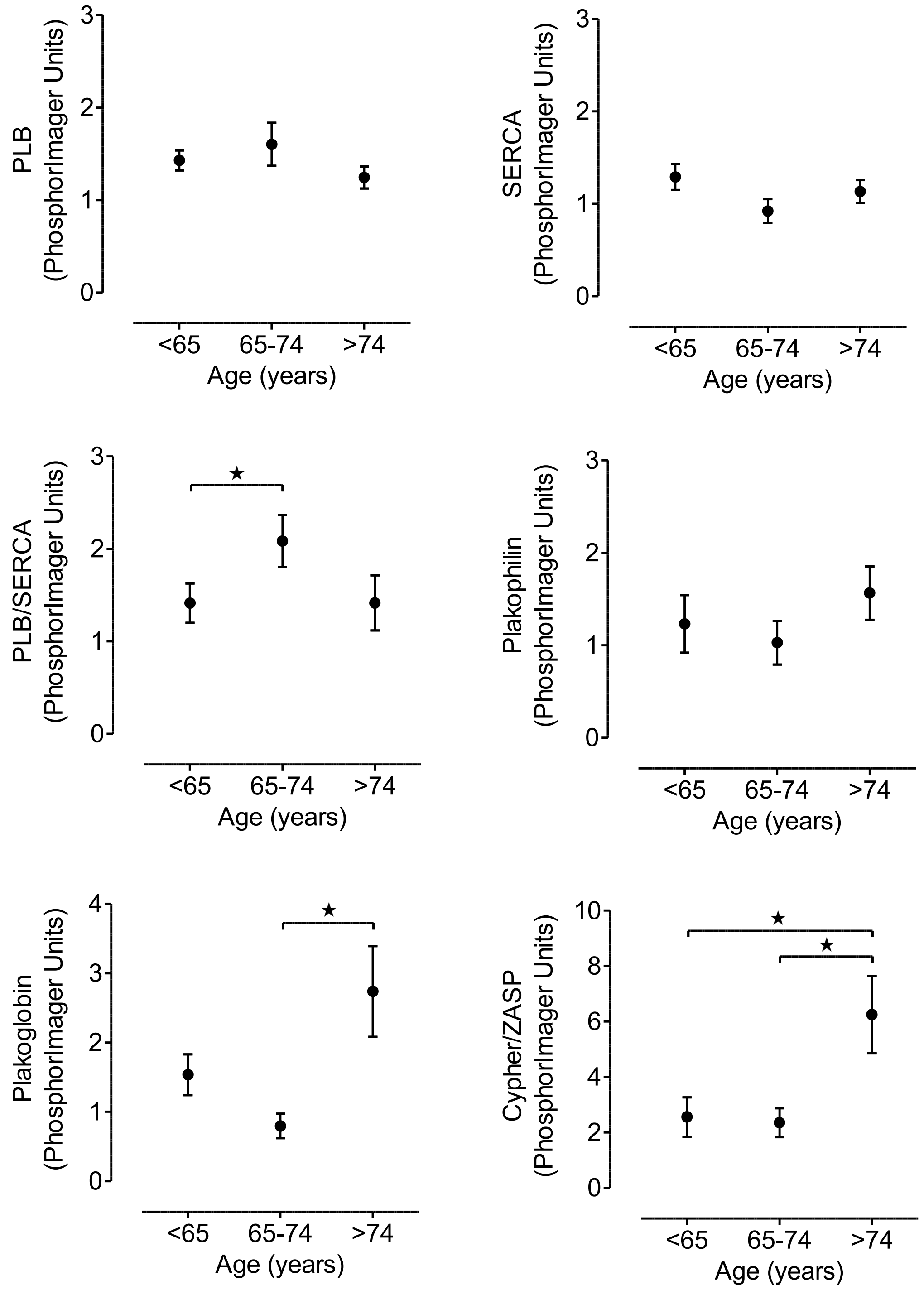

Figure S3. Expression of phospholamban (PLB), sarcoplasmic reticulum Ca^{2+}-ATPase (SERCA), the ratio of PLB and SERCA (PLB/SERCA), plakophilin, plakoglobin, and the Z-band protein cypher 1 (Cypher/ZASP) in human right atrium. Protein expression data ± SEM are categorized into three commonly used intervals. Ordinates are protein expression, normalized to cardiac calsequestrin in arbitrary PhosphorImager units versus age (abscissae). ⋆indicates a significant difference ($p<0.05$) between age groups calculated by ANOVA followed by Bonferroni's multiple comparison test. N(total) = 53, N(<65) = 20, N(65-74) = 19, N(>74) = 14.

Supplementary Tables

Table S1: Characteristics of patients included in the study.

Age (years)	NYHA	EF (%)	CCS	Statins	ASS	ACE inhibitor	AT1 receptor antagonist	β-Blocker
48	II - III	55	2	1	1	1	0	Metoprolol
51	II - III	65	2	1	1	1	0	Bisoprolol
52	II	60	2	0	0	0	0	Metoprolol
53	II	57	2	1	0	1	0	Metoprolol
53	II	52	2	1	1	1	0	Metoprolol
53	II	72	2	0	1	0	1	Metoprolol
56	II	65	1-2	1	1	1	0	Metoprolol
56	II	58	3	1	1	1	0	Metoprolol
57	II	76	3	1	0	1	0	Metoprolol
57	II	50	3	1	1	1	0	Bisoprolol
57	II	67	2	0	1	0	0	Bisoprolol
58	III	63	2	1	0	0	0	Metoprolol
60	II	60	2	1	1	1	0	Metoprolol
60	II	80	2	1	1	1	0	Bisoprolol
60	I	78	2-3	0	1	1	0	Bisoprolol
61	II	63	2	0	1	1	0	Metoprolol
62	III	40	2	1	0	1	0	Metoprolol
63	II	60	2	1	1	0	0	Metoprolol
63	II	70	2	1	1	1	0	Metoprolol
63	II	89	2	1	0	0	1	Metoprolol
65	II - III	55	2-3	1	1	1	0	Metoprolol
65	II	55	2	1	0	0	0	Metoprolol
65	II	71	3	0	1	1	0	Metoprolol
66	II	61	4	1	0	0	0	Bisoprolol
67	II	58	2	1	1	1	0	Bisoprolol
67	II	78	2	0	1	0	0	Metoprolol
68	II	35	2	0	1	1	0	Metoprolol
69	II	71	2	0	1	1	0	Carvedilol
69	II	72	2	1	1	0	0	Bisoprolol
70	II	75	2	1	1	1	0	Metoprolol
70	III	58	2	1	1	1	0	Metoprolol
70	II	65	3	1	1	0	0	Bisoprolol
70	II	74	2	1	1	1	0	Metoprolol
71	II	66	2-3	0	0	0	1	Metoprolol
73	II	62	2	1	0	0	0	Carvedilol
73	II	60	2	1	0	1	0	Metoprolol
73	II	46	2	1	0	0	0	Metoprolol
73	II	73	2	1	1	1	0	Metoprolol
74	II - III	35	2-3	1	1	1	0	Metoprolol
75	II	49	2	1	1	1	0	Metoprolol
75	II	44	2	1	1	0	1	Carvedilol
75	II	75	2-3	1	1	1	0	Metoprolol
76	I	74	2	0	0	1	0	Bisoprolol
76	II	70	2	1	1	1	0	Bisoprolol
77	III	50	2	1	1	1	0	Metoprolol
77	II	65	4	0	0	1	0	Metoprolol
78	II	55	2	1	1	1	0	Metoprolol
79	II	62	3	1	1	1	0	Carvedilol
80	I	71	1	1	0	1	0	Metoprolol
80	II	70	3	0	1	1	0	Bisoprolol
81	II	60	2	1	1	0	0	Bisoprolol
82	II	56	2	0	1	1	0	Metoprolol
84	I	60	4	1	1	1	0	Metoprolol

NYHA, New York Heart Association classification of heart failure
EF, left ventricular ejection fraction in %
CCS, Canadian Cardiovascular Society angina class
ASS, acetylsalicylic acid
ACE, angiotensin-converting enzyme
AT1 receptor, angiotensin II receptor type 1
In the columns for medications, "1" means yes and "0" means no.
